# Integrative p53, micro-RNA and Cathepsin Protease Co-Regulatory Expression Networks in Cancer

**DOI:** 10.3390/cancers12113454

**Published:** 2020-11-20

**Authors:** Surinder M. Soond, Maria V. Kozhevnikova, Paul A. Townsend, Andrey A. Zamyatnin

**Affiliations:** 1 Institute of Molecular Medicine, Sechenov First Moscow State Medical University, Trubetskaya str. 8-2, 119991 Moscow, Russia; 2 Hospital Therapy Department No. 1, Sechenov First Moscow State Medical University, 6-1 Bolshaya Pirogovskaya str, 119991 Moscow, Russia; Kozhevnikova-m@inbox.ru; 3 Division of Cancer Sciences and Manchester Cancer Research Centre, Faculty of Biology, Medicine and Health, University of Manchester, Manchester M13 9PL, UK; paul.townsend@manchester.ac.uk; 4 Belozersky Institute of Physico-Chemical Biology, Lomonosov Moscow State University, 119992 Moscow, Russia; 5 Department of Biotechnology, Sirius University of Science and Technology, 1 Olympic Ave, 354340 Sochi, Russia

**Keywords:** p53, cathepsin, micro-RNA, miRNA, cancer

## Abstract

**Simple Summary:**

This article describes an emerging area of significant interest in cancer and cell death and the relationships shared by these through the transcriptional regulation of cathepsin protease genes by micro-RNAs that are connected to p53 activation. While it has been demonstrated that the p53 protein can directly regulate some cathepsin genes and the expression of their upstream regulatory micro-RNAs, very little is known about what input the p53 isoform proteins may have in regulating this relationship. Herein, we draw attention to this important regulatory aspect in the context of describing mechanisms that are being established for the micro-RNA regulation of cathepsin protease genes and their collective use in diagnostic or prognostic assays.

**Abstract:**

As the direct regulatory role of p53 and some of its isoform proteins are becoming established in modulating gene expression in cancer research, another aspect of this mode of gene regulation that has captured significant interest over the years is the mechanistic interplay between p53 and micro-RNA transcriptional regulation. The input of this into modulating gene expression for some of the cathepsin family members has been viewed as carrying noticeable importance based on their biological effects during normal cellular homeostasis and cancer progression. While this area is still in its infancy in relation to general cathepsin gene regulation, we review the current p53-regulated micro-RNAs that are generating significant interest through their regulation of cathepsin proteases, thereby strengthening the link between activated p53 forms and cathepsin gene regulation. Additionally, we extend our understanding of this developing relationship to how such micro-RNAs are being utilized as diagnostic or prognostic tools and highlight their future uses in conjunction with cathepsin gene expression as potential biomarkers within a clinical setting.

## 1. Introduction

**Figure 1:**
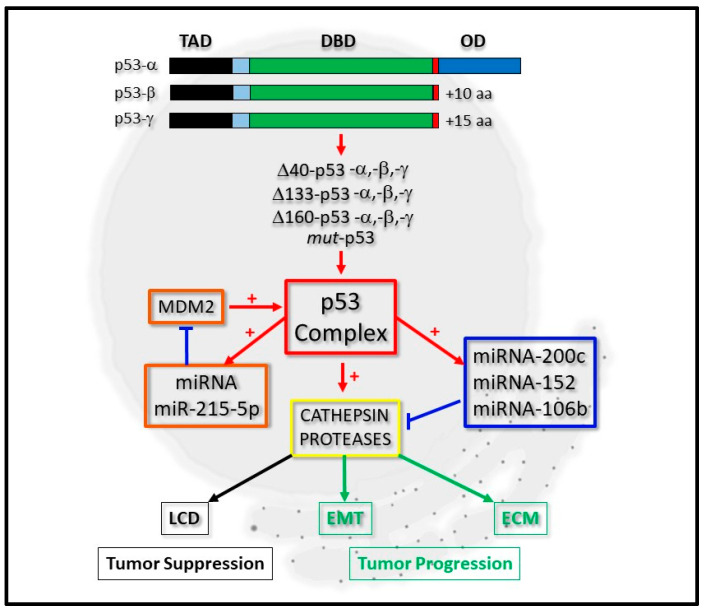
Integrative regulation of cathepsin proteases by p53 and micro-RNA expression. P53-alpha (p53-α) can be expressed as p53-beta (p53-β) or p53-gamma (p53-γ) isoform proteins, which lack the oligomerization domain (OD). Derivatives of these, which lack the complete Transactivation domain (TAD), but retain the DNA-binding domain (DBD), can also be expressed as Δ40-p53, Δ133-p53, Δ160-p53 or *mut*-p53 forms. The p53 complex can be regulated by micro-RNA (miRNA) expression through a positive feedback loop by positively regulating miRNA-215-5p, which negatively regulates MDM2 protein levels (orange boxes). It can transcriptionally regulate cathepsin protease expression directly or indirectly through directly regulating the expression of miRNA-200c, for example (blue box). Cathepsin protease expression (yellow box) contributes to lysosomal-mediated cell death (LCD) as a tumor suppressor (black boxes) or cell differentiation through Epithelial-Mesenchymal Transition (EMT) and the Extracellular Matrix (ECM) during tumor progression (green boxes).

The tumor suppressor gene *TP53* is mutated at a high frequency in a whole range of malignant diseases and has therefore been intensely researched for many years [1]. As is to be expected, the number of molecular networks that it has been shown to fundamentally regulate have also grown with great diversity and include aspects of DNA repair [2], cell senescence [3], angiogenesis [4], apoptosis [5,6] and cell cycle regulation [7]. While the main role of p53 in most of these processes are through its being able to directly regulate gene expression upon DNA binding, it can also mediate this through interacting with other transcription factors and regulators [8]. In some of its genetically mutated forms (*mut*-p53), p53 can take on the properties of a protein that is oncogenic, while some mutated derivatives can simply be inactive at the genetic or protein level [9]. Similarly, one key contributing factor originates from p53 being expressed as isoform proteins arising from the use of alternative promoters, translation initiation sites and mRNA splicing sites and which can act individually or in concert in modulating gene expression (Figure 1) [10,11].

From a regulatory perspective, p53 protein levels are kept to a minimum, through its polyubiquitination and destabilization by MDM2 and the proteasomal degradation pathway [9,10,11]. However, upon treating mammalian cells with oxidative stress or cytotoxic agents, nuclear p53 can become stabilized and modulate gene expression of proteins central to mediating cell arrest, DNA repair or apoptosis [12,13,14,15]. Additionally, post-translational modifications can also regulate p53 activity that mechanistically contribute to its cytoplasmic translocation, and where it can mediate mitochondria- or lysosomal-mediated cell death [16,17].

With over 14000 micro-RNAs annotated from the human genome that can regulate as much as 30% of all mRNAs expressed intracellularly, it is interesting to note that over 46% micro-RNA promoters have been reported to contain putative p53 binding sites [12,13]. While this highlights a potential direct link between p53 protein activation and micro-RNA expression, another important and direct role for the p53 protein in miRNA processing has also emerged. Here, p53 (or transcriptionally inactive p53) was revealed to be a central regulator of micro-RNA processing, through its ability to modulate the maturation of the micro-RNAs and their accessibility to mature mRNA messengers through its association with the protein Drosha [14] and the RISC complex (reviewed in [15,16]). Of importance is the ubiquitin ligase MDM2, which is under micro-RNA-mediated control as seen through the inhibitory actions of miRNA-192, miRNA-194, miRNA-215, miRNA-143, miRNA-145, and miRNA-605 expression [17]. For example, loss of miRNA-215-5p expression can enhance expression of MDM2, which results in diminished p53 protein levels [18]. As reported therein, p53 also positively regulated miRNA-215-5p expression, highlighting the existence of a p53 positive feedback loop [18]. Similarly, another good example of p53 regulation, through a miRNA acting on upstream activators of p53, occurs through miRNA-34, which acts by down-regulating the expression of the SIRT1 and HDAC intermediates that negatively-regulate p53 through its deacetylation (reviewed in [19]). While the actions of such micro-RNAs may give rise to enhanced levels of active p53 protein at the transcription and translation levels indirectly, p53 transcripts can also be directly targeted by miRNA-25 and miRNA-125b expression (reviewed in [16]).

The cathepsin proteases are a family of proteins that are developing greater importance due to them being intimately linked to tumor progression [20,21] and suppression [22]. During cancer progression, not only do they modulate the extracellular matrix and permit the dispersal of tumor cells following tumor growth, some of them also modulate the *trans*-differentiation of cells through the process of Epithelial Mesenchymal Transition (EMT) [21,23]. Simultaneously, the transcriptional regulation of cathepsins by p53 is also an area of research that is gaining much attention [22,24], particularly as lysosomes become more prone to lysis by lysosomorphic and cytotoxic agents upon cathepsin over-expression [25,26] and through p53 directly modulating lysosomal-mediated cell death [27,28], (Figure 1).

Consequently, the scientific interests revolving around the regulatory axis shared by all forms of p53, micro-RNAs and the cathepsins have captured the attention of many basic researchers, with a view to defining their co-regulatory relationships in greater depth (Figure 1). Herein, we review the recent progress that has been made in this area of research from an integrative perspective with a focus on how individual components of this regulatory axis may be explored further in a clinical setting.

## 2. The Biochemical Significance of the p53 Isoform Proteins

The p53 protein was first described over 30 years ago and its biological significance since then has had a significant amount of input into many of the p53-related paradigms that have been developed in many aspects of cancer cell biology. During this time, the *TP53* gene has also revealed itself to encode a number of important p53 isoforms proteins [10,11], which have set many precedents while laying a number of very strong foundations for the characterization of the subsequently discovered p53 somatic mutations with relative ease [9]. For simplicity, the p53 isoforms can be categorized into two groups (Figure 1). The first group contains the p53-α, p53-β and p53-γ forms (which respectively encode WT-p53 (wild-type p53) and isoforms lacking the carboxyl-terminal Oligomerization Domain (OD), which is replaced with 10–15 amino acid extensions formed through alternative splicing of the mRNA (Figure 1 and Table 1). While these are driven transcriptionally from the promoter upstream of the first exon [29], Δ40-p53 isoform derivatives can also arise through the alternative splicing of the p53 transcript and the use of the initiator AUG at codon 40 [30]. Additional p53 protein derivatives (lacking part of its amino-terminal) can also arise from transcripts being driven from a second promoter located between intron 1 and exon 5, giving rise to ΔN-terminal p53 isoforms which have a 133 and 160 amino acid deletion at the amino-terminal [30,31,32]. Broadly, the p53 derivatives lacking the amino termini can be categorized into the second group (Figure 1 and Table 1).

Biologically, all of the p53 isoforms exhibit diverse degrees of dominant-inhibitory effects for *trans*-activating gene expression through their abilities to form *homo*-tetramers or *hetero*-tetramers with WT-p53 [30,31,34]. This is based upon some of the isoforms lacking the OD, the full *trans*-activating domain (TAD) and showing varying degrees of protein stability and transcriptional activity based on the presence or absence of key phosphorylation sites, such as Ser-46 [35,36,37,38] and the carboxyl-terminal MDM2-specific ubiquitination sites [34,39]. Importantly, their biochemical characterization has indeed helped in offering an insight into how the p53 proteins arising from somatic mutations within the *TP53* gene may differ biochemically in comparison to WT-p53 (or p53-α). Such mutations can be broadly described as a gain of function (GOF) or a loss of function (LOF) and the most commonest of them are the R175, G245, R248, R249, R273 and R282 mutants (collectively known as *mut*-p53) and which make up around 30% of all mutations found within the *TP53* gene [40,41,42].

More specifically, the characterization of such p53 mutants has offered some excellent mechanistic insights into how certain micro-RNAs are regulated transcriptionally, especially in the context of cancer progression. For example, as far back as 2011, Chang et al. (2011) reported that miRNA-200c expression could be down-regulated upon the expression of a number of *mut*-p53 derivatives in 106 patient samples and MCF12A BC cells, which correlated significantly with tumor grade [43]. More recently, the expression of *mut*-p53 has also been linked to decreased miRNA-200c expression in human osteosarcoma cells by Tamura et al. (2015, [44]) and Alam et al. (2017, [45]) who identified the R280K *mut*-p53 protein as being responsible for this [45]. Here, increased expression levels of Moesin in MCF7 1001 BC cells were reported, as a significant contributing factor to carcinogenesis.

Collectively, the existence of such a high number of p53 isoform proteins can potentially offer a number of alternative mechanisms for how the *TP53* gene can exert its biological effects. Consequently, their importance in being able to regulate tumor suppressive miRNA expression, either exclusively or with WT-p53, is being viewed as mechanistically significant during tumor initiation or progression.

## 3. p53, micro-RNA Regulation and Cathepsin Proteases: A Developing Network

The family of cathepsin proteases is composed of aspartate proteases (D, E), serine proteases (A, G) and the cysteine proteases (B, C, F, H, K, L, O, S, V, Z/X, W) [20]. Collectively, they are expressed as inactive zymogens, which have the capability to become *auto*-activated or *trans*-activated as they traffic from the endosome to reside within the lysosome, but can also be found in the nucleus [46]. Some of them are upregulated in expression, especially during cancer progression and can be secreted into the Extracellular Matrix (ECM) where they can modulate ECM components and contribute to malignancy [47,48]. Nevertheless, normally they are localized within the lysosome, from where they can leak into the cytoplasm and activate intermediates from the intrinsic apoptotic pathway as in the case of BID cleavage, causing the activation of apoptosis [49].

More recently, cathepsins L and D have been seen to reside in the nucleus where they can cleave the Histone H3 protein [50,51], CUX1 [52,53,54], TRPS1 [23] and enhance proliferation, induce EMT and increase the motility of cells. Consequently, a strong interest in how cathepsin expression is regulated has developed with the transcriptional regulation of cathepsins D and L having been linked to p53. Here, cathepsin D was expressed in a p53-dependent manner in U1752, Pa1 and ML1 leukemia cell manner and p53 was reported to bind to two p53 consensus sequences within the cathepsin D promoter [22]. Similarly, p53 could bind the promoter region of cathepsin L and the expression of which could also be driven by *mut*-p53 expression in glioblastoma cells [24]. Being mindful of these observations, there are justifiable reasons for why the scope of research here needs to be broadened in order to ascertain how cathepsins may be regulated in the absence and presence of p53 (or its isoforms and *mut*-p53 derivatives), and whether such events can still permit the cathepsins to drive tumor progression.

Generally speaking, developing interests have revolved around how cathepsin genes may be regulated by specific micro-RNAs, and of importance here is how these may be linked to what is commonly known about p53 and cathepsin protease regulation. In the instance of cathepsin proteases, this area of research appears to be relatively undeveloped, and being mindful of there being around 15 cathepsin proteases (with the majority of them being linked to cancer development or progression [20]), reportedly only a few of them appear to be regulated by micro-RNAs that have a direct or indirect connection with p53. Moreover, the regulation of cathepsins in the context of p53 isoforms or mutant-derivatives thereof appear to be even less explored and is an important consideration in light of how quickly this area of p53 biology is expanding.

Consequently, in highlighting the nature of these developing integrative regulatory networks, the next section is devoted to reviewing, a) which micro-RNAs are regulated by (or regulate) p53, and b) how these micro-RNAs regulate cathepsin protease family members in the context of cancer, with a view to broadening our understanding of the regulatory interplay between p53 and cathepsin transcription. Broadly speaking, miRNA-200c, miRNA-152 and miRNA-106b appear to be the most characterized in this context, with others such as miRNA-29a (cathepsin K, [55]), miRNA-30 (cathepsin D, [56]), miRNA-25-3p (cathepsin K, [57]), miRNA-140 (cathepsin B, [58]), miRNA-483-5p (cathepsin K, [59]) and miRNA-506-3p (cathepsin K, [60]) being characterized to a lesser extent (Table 2).

### 3.1. miRNA-200c and Cathepsin Regulation

MiRNA-200c originates from the miRNA-200 family of micro-RNAs, composed of miRNA-200a, miRNA-200b, miRNA-200c, miRNA-141 and miRNA-429 [66,67]. Their importance is emerging as being significant in many biological processes such as EMT, cell invasion, proliferation, metastasis, apoptosis, autophagy and therapy resistance in several cancer types [68,69,70,71,72]. miRNA-200c forms part of the miRNA-200c-25 cluster encoded on chromosome 12 [73,74] and has gained particular importance due to its contribution to tumorigenesis, chemoresistance, migration and stemness [75]. At the molecular level, it shares a very close relationship with p53 expression as it can be positively regulated by it [43] and in doing so, can negatively regulate EMT [76] and tumor aggressiveness [77].

Downstream, miRNA-200c expression negatively regulates its target gene *ZEB1* [43], which usually suppresses critically important EMT-regulatory gene products [74,78,79,80,81] such as E-cadherin [82] during tumor invasiveness and stemness [43]. Consequently, upon the loss of active p53, miRNA-200c expression is reduced and gene suppression by Zeb1 enhanced, causing the loss of E-cadherin expression [43,83]. In keeping with p53 activation, upon oxidative stress of cells, miRNA-200c can also contribute to cellular senescence and apoptosis of human umbilical vein endothelial cells [84] and has been found to be increased in expression during colorectal cancer (CRC) progression while reportedly also serving usefulness as a prognostic marker [85].

One cathepsin protease family member found to be categorically important in modulating EMT is cathepsin L, through its ability to translocate to the nucleus in prostate, breast, lung cancer and glioma cells and cleave to Histone H3 and CUX1 proteins resulting in the onset of EMT [53,54,86] (reviewed in [46]). Enhanced cathepsin L promoter activity through a p53-dependent manner has also been documented in glioblastoma cells [24], U251 [87] and non-small cell lung cancer cells [88] in addition to being connected with miRNA-200c regulation [61]. Here, enhanced miRNA-200c expression led to decreased levels of cathepsin L expression and sensitization of cells to paclitaxel-mediated apoptosis in A549 lung cancer cells [61]. Cathepsin L knockdown was observed to increase miRNA-200c expression and overexpression of cathepsin L could reverse this effect [61]. Moreover, inhibition of miRNA-200c, enhanced cathepsin L levels, which collectively suggest the existence of a regulatory feedback loop. Taken with miRNA-200c modulating EMT through *ZEB1* suppression, expression of cathepsin L was able to permit EMT progression, as seen upon interfering with miRNA-200c expression. Taking into consideration that p53 expression may enhance transcriptional activation of cathepsin L expression, these findings suggest a mechanism whereby EMT can be induced through the down-regulation of *ZEB1* (upon cathepsin L expression) in a p53-dependent manner. Such a mechanism could also be extended in explaining how the loss of miRNA-200c contributes to tumor progression and which might have greater biological significance if correlated with cathepsin L expression levels [61,79,89].

Collectively, such studies highlight the interplay of p53, micro-RNA and cathepsins as an important basis for the modulation of EMT in cancer progression. With certainty, the regulatory networks shared by cathepsin L in promoting EMT and tumor progression, through the loss of miRNA-200c expression does appear to be offering some clarity as to how cathepsin L expression may still be able to mediate EMT in the absence of p53 driving its transcriptional expression or in the presence of *mut*-p53 expression.

### 3.2. miRNA-152-3p and Cathepsin Regulation

As a recent development, miRNA-152 expression was reported to be elevated upon Ionizing Radiation (IR) treatments and during the senescence of WI38 cells, while being seen to be decreased in expression within cells transformed with the SV40 Large-T antigen [90]. The importance of this micro-RNA comes into focus as a downstream effector of p53 and the tumor protein 53-induced nuclear protein 1 (TP53INP1), the expression of which likely regulates phospor-p53-dependent apoptosis by serving as a co-factor for the putative p53-Ser46 kinase [62]. Through using micro-RNA profiling in U251 cells, miRNA-152-3p levels were also observed to be regulated and increased in response to Glial cell line-Derived Neurotrophic Factor, GDNF [91], which caused downregulation of Desmocollin-2 (DSC2) expression and was seen to be correlated with increased tumor grade [92,93]. Mechanistically, miRNA-152-3p expression can also be regulated by epigenetic changes, as seen in its decrease expression upon micro-RNA promoter methylation in endometrial cancers [94], cholangiocarcinoma and gastrointestinal cancer [95,96,97]. Clearly, such observations highlight a connection between p53 and miRNA-152-3p expression and the regulatory nature of this under IR and GDNF stimulatory conditions, where the methylation status of its promoter region may also be of emerging significance. More recently, miRNA-152-3p has been linked to targeting cathepsin L transcriptional suppression, resulting in reduced cellular migratory capacity, enhanced cell cycle arrest and apoptosis of a number of gastrointestinal stromal cell lines and normal RGM-1 cells [63].

Collectively, the picture emerging appears to highlight the importance of p53-mediated miRNA-152-3p expression (probably through TP53INP1 expression) and which appears to have the effect of indirectly reducing cathepsin L gene expression and the biological effects associated with its expression.

### 3.3. miRNA-106b and Cathepsin Regulation

The amplification of chromosome 7q21-22 has been reported in leukemia, gastric, oesophageal, liver, prostate and endometrial cancers [98,99,100,101,102,103]. This region encodes two poly-cistronic micro-RNA clusters (miRNA-106-25 and miRNA-25) encoded within intron 13 of the MCM7 gene [104] giving rise to increased miRNA-106b expression in chronic lymphocytic leukemia patients [105]. Similarly, miRNA-106b expression has also been linked with glioma [106], prostate [100], gastric [107] and hepatic cancer progression [101,108]. Mechanistically, miRNA-106b can negatively interfere with p21 expression [98,109,110] and thereby modulate cell proliferation and cell survival, particularly during the DNA damage response [100,101]. Of additional importance is p53 expression, as it can downregulate the miRNA-106b cluster [111] by repressing E2F1 activity through BTG3 protein-mediated inhibition [112]. Moreover, inhibition of miRNA-106b through the use of antagomers can enhance p53 promoter and protein activity in renal clear cell carcinoma cells, highlighting the importance of miRNA106-b expression in a possible p53-directed negative regulatory loop [113].

Cathepsin A expression is important in malignant melanoma and CRC progression, and has been linked to p53 expression through the effects of miRNA-106b expression [64]. In a recent study, miRNA-106b expression was observed to be decreased in CRC tissue samples and increasing miRNA-106b expression found to suppress migration and the invasiveness of CRC cells [65]. Importantly, miRNA-106b could directly bind to the 3’ UTR of the cathepsin A mRNA, causing a decrease in cathepsin A protein expression [65]. Collectively, the relationship that appears to be emerging suggests that while amplified miRNA-106b expression might contribute to cancer progression in a cell-type context manner, in CRC metastases its expression appears to be suppressed, giving rise to enhanced levels of cathepsin A expression during CRC metastasis.

In summary, the regulation of cathepsin L by miRNA-200c appears to be far more developed than the work reported for miRNA-106b and miRNA-152-3p, based on the relationship it shares with p53-mediated transcriptional regulation and how EMT is modulated (in light of this) by miRNA-200c and Zeb1 as co-regulators. In this scenario, however, very limited research has been conducted in the context of which p53 isoforms may regulate the outlined mechanisms for direct cathepsin regulation or indirect regulation through miRNA-200c regulation and future experiments may help to address this.

## 4. miRNA-200c, -152, -106b Expression and Cancer Progression: A Clinical Perspective

Based on the above, there are clear regulatory relationships that are emerging between p53, cathepsin and micro-RNA expression. While the focus of this article has so far been originated from defining the molecular roles that p53 and cathepsins share in disease progression, for completeness we would like to extend the importance of the above miRNAs within a clinical context. This has great significance through the common biological traits their downstream target gene products share with some of the cathepsin proteases, and therefore it is worth focusing on this through highlighting alternative transcripts (or proteins) that are targeted by these miRNAs. In addition to this, we would like to review the recent progress on how these micro-RNAs are being utilized in diagnostic and prognostic assays. For simplicity and consistency, we will keep the focus and the context as close to lung cancer (miRNA-200c), gastric cancer (miRNA-152) and colorectal cancer (miRNA-106b), as possible.

### 4.1. miRNA-200c Expression

As far back as 2013, the importance of miRNA-200c in the regulation of disease progression has positively been gaining greater momentum. For example, the loss of miRNA-200c within the lungs [114] was seen to correlate with NSCLC cells showing an invasive and chemo-resistant phenotype [115], while positive expression of it could sensitize cells to chemotherapeutic [116] and radiotherapeutic [117] agents. As reported by Cortez et al. (2014), such findings could be extended and they reported the expression of miRNA-200c enhanced radio-sensitivity of cells in a xenograft lung cancer model through miRNA-200c expression inducing the oxidative stress response by its regulation of oxidative response genes [118]. Similarly, Shi et al. (2013) showed that A549 cells could be radio-sensitized upon miRNA-200c expression [117], while Kopp et al. (2013) showed that miRNA-200c could target K-Ras expression and that it could inhibit tumor progression and therapeutic resistance in a panel of BC cell lines [119]. Additional tumor suppressive effects have also been reported and which showed miRNA-200c expression to decrease NCCLC and A549 migration or invasiveness. MiRNA-200c was also reported to target *USP25* [120], *ZEB1* [121] or *ZEB2* [122], and had the effect of modulating cell migration and differentiation of cells. Similarly, miRNA-200c expression was also correlated with reduced cell migration of H23 cells through enhanced E-cadherin expression [123]. Conversely, miRNA-200c was also seen to function by inducing cell death through the apoptotic pathway. For example, Bai et al. (2014) showed that miRNA-200c expression targeted the RECK gene and induced the apoptotic death of H460 lung cells, which was enhanced in the presence of Reservatol stimulation [124]. Generally, the functional role of positive miRNA-200c expression appears to be one that minimizes tumor progression and is mechanistically linked to the suppression of genes that have an oncogenic effect (Table 3).

Simultaneously, a number of excellent studies have also published how miRNA evaluation in cells can be successfully utilized as a diagnostic and prognostic tool. For example, Tejero et al. (2014) reported that miRNA-200c could be a good biomarker for overall survival (OS) during the early stages of NSCLC adenocarcinoma [123]. Here, qRT-PCR was used to evaluate 155 resected patient tumor samples for miRNA-200c expression and their findings complimented with functional studies using H23, HCC44 and A549 cell lines. Elevated miRNA-200c expression in early stage NSCLC was significantly correlated with a decrease in OS [123]. Similarly, Kim et al. (2014) reported miRNA-200c expression to be significantly up-regulated and correlated with tumor size, lymphovascular invasion and poor OS [125]. Other publications supporting such trends have also been recently reported through the extensive use of meta-analyses to help define the diagnostic potential of miRNA-200c expression. For example, Shao et al. (2015) correlated high levels of circulating miRNA-200c with a poor OS and PFS (in advanced disease) and low miRNA-200c levels with poor survival during early stages of disease [126]. Here, 18 published studies were analyzed and the regulation of EMT (or MET) by miRNA-200c was seen as a possible cause. Teng et al. (2016) identified circulating and tissue-derived miRNA-200c as a potential diagnostic and prognostic marker for epithelial ovarian cancer (EOC) [127]. Si et al. (2017) analyzed 110 resected tumor samples from NSCLC patients for quantification of miRNA-200c, the expression of which was associated with positive lymph node metastasis, TNM classification and a reduced 5 year disease-free survival rate [128]. More recently, the use of miRNA as biomarkers for responsiveness to chemotherapeutics have also gained some attention as reported by Li et al. (2017). Here, the findings from 46 published articles showed that low expression levels of miRNA-200c (or IHC negative staining) was a good predictor for responsiveness to chemo- or radio-therapy in esophageal cancer [129]. Moreover, Zheng et al. (2017) used a meta-analysis from 60 reported studies to highlight that increased miRNA-200c expression correlated with poor prognosis in gastrointestinal cancer (GIC) patients [130], while increased miRNA-200c expression offered a better OS for ovarian cancer (OC) patients, as reported by Shi et al. (2018) [131], (Table 4).

### 4.2. miRNA-152 Expression

The expression of miRNA-152 has been evaluated in a number of cancers associated with the gastrointestinal tract over the last 10 years with some very clear findings on which target genes may be regulated by miRNA-152 and what role they may play during cancer progression. For example, Chen et al. (2010) analyzed 101 gastric cancer (GC) and colorectal cancer (CRC) tissue samples and reported a decrease in miRNA-152 expression, which correlated with an increased tumor size and advanced pT stage in GIC, and inversely correlated with cholecystokinin B receptor protein expression in GC [96]. Other target genes for miRNA-152 include PIK3CA in breast cancer (BC) [132] or PIK3R3 in CRC [133], EPAS1 in Paclitaxel-resistant BC cells [134], CD151 in GC [135], IGF-1R and IRS1 in BC [136], B7-H1 in GC [137], CDK8 in hepatocellular carcinoma (HCC) [138], p27 in bone marrow cells [139], SOS1 in Glioblastoma (GBM)[140] cells and KLF4 in colon cancer (CC) cells [141], (Table 5).

At the clinical level, Safrinzo et al. (2013) showed stage I-IIIA NSCLC patient plasma samples to contain decreased miRNA-152 expression levels, which correlated with decreased DFS for lung squamous cell carcinoma prevalence (SCC) [142]. Li et al. (2016) reported a decrease in expression of miRNA-152 in CRC tissues which inversely correlated with TNM staging and lymph node metastases [133], while Wang et al. (2017) observed a decrease in miRNA-152 expression in GC patients [137] and Ge et al. (2017) showed that miRNA-152-3p could target PIK3CA in BC as a tumor suppressor [132]. You et al. (2018) analyzed 15 GC tissues and confirmed that miRNA-152-3p expression was reduced and could directly target PIK3CA in SGC-7901 cells [143]. Alternatively, Matin et al. (2018) profiled 372 patient plasma samples collected before, during and after treatments for PC and elevated miRNA-152-3p levels reported, while (interestingly) low levels of miRNA-152-3p expression were observed in prostate cancer (PC) samples [144]. Such findings indeed highlight the power of miRNA-152 quantification as a diagnostic marker for PC (as seen for lung cancer, CRC and BC [145]). In CML, miRNA-152-3p expression was elevated in bone marrow (BM) samples and upon expression of miRNA-152-3p in K562 cells, proliferation was decreased and apoptosis levels were enhanced through targeting the p27 (*CDKN1B*) gene [139]. From the analysis of 89 HCC tumor samples, Yin et al. (2019) showed that miRNA-152-3p levels were decreased and which correlated with tumor volume and TNM staging [138]. Moreover, Wang et al. (2017) saw that decreased miRNA-152 expression was related to poor OS and DFS in GC, which could be used as an independent risk factor for the prediction of HCC prognosis [137]. More recently, Li et al. (2019) diagnosed early stage I-II BC by screening 106 plasma samples and tissues for miRNA-152-3p expression and reported it to be decreased, which correlated with ER-positive and PR-positive patients [146]. Finally, Song et al. (2020) observed reduced levels of miRNA-152-3p in a study of 30 invasive BC samples, which correlated with a poor prognosis [134] and the overexpression of which could sensitize chemo-resistant BC cells to Paclitaxel-mediated cell death (Table 6).

### 4.3. miRNA-106b Expression

While miRNA-106b expression has indeed emerged as having a tangible biological effect in most cancer cell systems, the outcomes from such studies at this moment have offered mixed results and appears to be an area of research development. Cai et al. (2011) reported that miRNA-106b could target RB expression in laryngeal carcinoma [147] and ATG16L1 expression in Crohn’s Disease samples [148,149]. Additionally, all three micro-RNAs from the miRNA-106b-25 cluster were seen to target PTEN expression [150,151] and increased miRNA-106b expression recorded in CRC tissues which could target DLC-1 (while enhancing EMT, [152]) and FAT4 in CRC tissues or cell lines [153], (Table 7).

Based on the growing importance of utilizing miRNA expression within the clinic, their quantification for the diagnosis and prognosis of patients has moved in a positive direction. In the instance of miRNA-106b a number of excellent studies have significantly shaped this area and are worth mentioning.

As far back as 2010, Wang et al. (2010) analyzed CRC samples using qRT-PCR and found miRNA-106b to be up-regulated [154] as confirmed thereafter in colorectal cancer stromal tissues as well [155]. Subsequently, Wang et al. (2015) found miRNA-106b expression to be increased in 180 CRC patients, which correlated with a longer OS but were not seen as being statistically significant [156]. Similarly, Zhang et al. (2015) analyzed 95 CRC patient samples and miRNA-106b expression correlated with a shorter OS or DFS and which had significant reliability as an independent prognostic factor for CRC [157]. In the context of RCCC, Gu et al. (2015) performed a meta-analysis on 27 studies analyzing the expression of miRNA-106b, and (unlike CRC) reported that a decreased miRNA-106b was associated with a poor prognosis [158]. More recently, high exosomal miRNA-106b levels from the serum have been reported to correlate with a high TNM stage, a larger tumor volume and a poor prognosis [152].

Collectively, such findings support the notion that the use of miRNA-106b as a prognostic marker is unreliable, based on inconsistencies reported from a number of studies correlating miRNA expression levels with tumor grade (Table 8).

In summary, relatively good progress is being made in defining target genes for the above specific miRNAs, which may help to offer a broader perspective on how other genes of importance may synergize with cathepsin regulation in disease progression. Moreover, additional insights are also emerging into how such micro-RNAs can be utilized as reliable diagnostic and prognostic markers to possibly compliment on-going efforts with other biomarkers of importance, such as p53 and cathepsin expression. Additionally, from the above studies, oncogenic micro-RNAs are also emerging to play an important regulatory role in disease progression, and do have the potential to be targeted for therapeutic purposes using small molecule-inhibitors or -degraders (as reviewed in [159]) or through targeting specific upstream transcription regulatory signaling pathways.

## 5. Future Perspectives

As reported herein, significant progress is being made in connecting p53 and micro-RNA expression with the transcriptional regulation of some cathepsin members from an “integrative” perspective. Clearly, by looking at the broader picture, we are better positioned to look at the finer details with regards to how such regulatory mechanisms may also be developed further in order to develop a clearer mechanistic picture of disease progression from the molecular to the biological level. However, in doing this, a number of fundamental questions do indeed come to mind. For example, “would the molecular mechanisms that have surfaced be any different in the presence of p53 isoform protein expression in place of WT-p53 expression?” Predictably, the answer to this question is likely “yes”, based on the isoform proteins derived from the *TP53* locus exhibiting very different biochemical properties in relation to WT-p53. Nevertheless, we must also be mindful that the areas of research we have discussed herein are still in a stage of infancy and the roles played by the p53 isoforms (or *mut*-p53 derivatives) in unveiling or furthering our understanding of cathepsins or specific micro-RNA gene regulation mechanisms should be given greater priority. While considering the importance of p53 isoforms, we must also not lose sight of the fact that some cathepsin proteases also exist as isoform proteins, derived from alternative splicing of their cognate mRNAs and the biological significance of which cannot be ignored. This is of particular importance in the instance of some cathepsin L isoforms proteins, which can translocate to the nucleus.

While great strides have been taken in assessing the input of some p53 isoforms or *mut*-p53 derivatives into how cells respond to stimulatory cues and modulate cell death, their clinical evaluation as biomarkers in conjunction with cathepsin proteases that drive tumor progression could also be a strong area for development in the near future. For example, a recent study by Guerra et al. (2016) touched upon the importance of detecting p53 mutant derivatives as co-biomarkers with cathepsin D expression in BC prognosis and which offered reliability as markers for ascertaining BC relapse [160]. When taken with cathepsin proteases as being attractive diagnostic and prognostic markers for cancer (reviewed in [21]), there is clearly great potential for such assays to possibly incorporate and evaluate the expression of cathepsin-specific micro-RNAs, to offer greater reliability and consistency. In particular, the use of quantitative-RT-PCR in evaluating serum-derived miRNAs of interest is revealing itself to be a useful and reliable clinical assay. While such serum-based protein assays for assessing cathepsin expression in disease (exclusively) may not necessarily offer a reliable readout, and may therefore rely on the use of classical IHC approaches, the addition of serum-based q-RT-PCR micro-RNA assays to compliment such approaches could be an interesting area for future exploration with good potential. Moreover, such classical IHC approaches for cathepsin protease evaluation could also incorporate the evaluation of their alternative target gene products that cathepsin-specific micro-RNAs have the potential to regulate, such as *ZEB1*. Such an approach takes great advantage of micro-RNAs having the ability to target more than one gene product and some of which can be evaluated and explored further in bioassay development.

## 6. Conclusions

Some p53 protein isoforms have indeed being given greater emphasis in shaping how we view the activities of WT-p53 within micro-RNA regulatory mechanisms and cathepsin gene expression, as seen for cathepsins L and A. Collectively, while this relationship is still developing at the molecular level, it has great potential to be explored further for other cathepsin proteases, in basic research and in a clinical context, when we view progress from a broader perspective. In this review article, we have merely touched upon how a selective number of regulatory networks and their protein intermediates can be co-modulated, in manner and form. As highlighted, regulation of p53 and the relationship it shares with micro-RNAs and their target genes is a very prolific area of research, in relation to the area of micro-RNA and cathepsin protease regulation. From a clinical perspective, this axis of cathepsin protease regulation and how it can be utilized for diagnostic and prognostic purposes does hold great potential and is also an area that is developing, albeit at a relatively slower pace. Nevertheless, there are some aspects of all three of these regulatory components that are slowly beginning to converge or overlap with some very scientifically promising findings being reported. While it is inevitable that such networks can develop complexity quite rapidly and may therefore offer some limitations, the contribution that such networks may offer, particularly in the context of personalized medicine, does highlight their emerging importance and significance. 

## Figures and Tables

**Table 1:** p53 isoform proteins.

p53 Isoform	Amino Acids	Protein (kD)	Reference
p53-α	1-393	53	[33]
p53-β	1-331+10	47	[10]
p53-γ	1-331+15	48	[29]
Δ40-p53-α	40-393	47	[30,31]
Δ40-p53-β	40-331+10	42	[29]
Δ40-p53-γ	40-331+15	42	[29]
Δ133-p53-α	133-393	35	[29]
Δ133-p53-β	133-331+10	29	[29]
Δ133-p53-γ	133-331+15	29	[29]
Δ160-p53-α	161-393	31	[32]
Δ160-p53-β	161-331+10	26	[11,32]
Δ160-p53-γ	161-331+15	26	[11,32]

The *TP53* gene can be transcriptionally driven by a second promoter, giving rise to p53 isoform proteins which lack regions of their amino terminal (Δ40-p53, Δ133-p53, Δ160-p53 proteins) and which can also lack the carboxyl-terminal ODs, as in the p53-αβ, p53-β and p53-γ isoforms. Their amino acid coding regions are highlighted, as are their predicted sizes.

**Table 2:** The developing networks between micro-RNA, cathepsin proteases and p53 expression.

Micro-RNA	Cathepsin	p53 Isoform	Cell Type	Reference
miRNA-200c	L	WT-p53-α	A549 Lung	[43,61]
miRNA-152	L	WT-p53-α	Gastrointestinal	[62,63]
miRNA-106b	A	WT-p53-α	Colorectal	[64,65]
miRNA-140	B	-	Glioblastoma	[58]
miRNA-30	D	-	Macrophage	[56]
miRNA-25-3p	K	-	Osteoblast	[57]
miRNA-483-5p	K	-	PBMC	[59]
miRNA-506-3p	K	-	Macrophage	[60]
miRNA-29a	K	-	Osteoblast	[55]

The expression of micro-RNAs connected with cathepsin gene expression are highlighted in conjunction with specific p53 isoforms and cell types they have been collectively characterized in (WT-p53, wild-type p53; PBMC, Peripheral Blood Mononuclear Cells; -, unknown).

**Table 3:** Elevated (+) or reduced (−) miRNA-200c levels are shown, as are their target genes, their biological effects and whether these factors can sensitize cells to certain therapeutic agents. The cell types indicate the types of cells characterized. BC, breast cancer; NSCLC, non-small cell lung cancer.

micro-RNA	Target	Negative Effect	SensitizingAgent	Cell Type	Reference
200c (+)	VEGF, VEGFR2	Angiogenesis, Cell Migration	Radiation	A549	[117]
200c (+)	PRDX2, SENS1, GABPA/Nrf2	Oxidative Response	Radiation	A549, H460, H1299	[118]
200c (+)	K-Ras	Proliferation, Cell cycle	−	Lung and BC cell lines	[119]
200c (+)	USP25	Cell Migration EMT	−	NSCLC cell lines	[120]
200c (−)	ZEB1	Cell Migration	Gefitinib	PC-9-ZD	[121]
200c (−)	ZEB2	EMT	−	A-549	[122]
200c (+)	Possibly E-cadherin	Cell Migration	−	H23, A549, HCC-44	[123]
200c (+)	Possibly RECK	Proliferation	Reservatol	H-460	[124]

**Table 4:** Elevated (+) or reduced (−) miRNA-200c levels are shown, as are the cancer types, source of materials the miRNA was detected from and the patient cohort size. NSCLC, non-small cell lung cancer; EOC, epithelial ovarian cancer; GIC, gastrointestinal cancer; esophageal cancer (ES); OC, ovarian cancer. The negative or positive use of the technique in diagnostic or prognostic evaluation of patients are denoted by − or +, respectively.

micro-RNA	Cancer Type	Source	Cohort Size	Diagnostic	Prognosis	Reference
200c (+)	NSCLC	Tissue	155	−	Reduced	[123]
200c (+)	NSCLC	Tissue	72	−	Reduced	[125]
200c (−)	varied	Tissue/Blood	18 studies	−	Poor OS and PFS	[126]
200c (+)	EOC	Tissue/Plasma	14 studies	+	+	[127]
200c (+)	NSCLC	Tissue	110	−	Reduced	[128]
200c (−)	EC	Tissue	46 studies	−	+	[129]
200c (+/−)	GIC	Tissue/Blood	60 studies	−	+	[130]
200c (+)	OC	Tissue/Blood	15 studies	−	+	[131]

**Table 5:** Elevated (+) or reduced (−) miRNA-152 levels are shown, as are their target genes, their biological effects and whether these factors can sensitize cells to certain therapeutic agents. The cell types indicate the types of cells characterized. BC, breast cancer; GC, gastric cancer; CRC, colorectal cancer; BM, bone marrow; GBM, glioblastoma; HCC, hepatocellular carcinoma; CC, colon cancer.

micro-RNA	Target	Negative Effect	Sensitizing Agent	Cell Type	Reference
152 (−)	PIK3CA	Cell Proliferation	−	HCC1806	[132]
152 (−)	PIK3R3	Cell Proliferation Migration	−	CRC cell lines	[133]
152 (−)	EPAS	Apoptosis	Paclitaxel	BC cell lines	[134]
152 (−)	CD151	Proliferation Migration	−	GC Tissues	[135]
152 (−)	IGF-1R	Proliferation Angiogenesis	−	BC Tissues	[136]
152 (−)	IRS1	Proliferation Angiogenesis	−	BC Tissues	[136]
152 (−)	B7-H1	T-cell Proliferation	−	GC cell lines	[137]
152 (−)	CDK8	Proliferation Apoptosis	−	HCC cell lines	[138]
152 (+)	p27	Proliferation	−	BM cells, K562	[139]
152 (−)	SOS1	Proliferation Apoptosis	Cisplatin	GBM cell lines	[140]
152 (+)	KLF4	Proliferation	−	CC cell lines	[141]

**Table 6:** Elevated (+) or reduced (−) miRNA-152 levels are shown, as are the cancer types, source of materials the miRNA was detected from and the patient cohort size. The negative or positive use of the technique in diagnostic or prognostic evaluation of patients are denoted by - or +, respectively. NSCLC, non-small lung cancer cells; CRC, colorectal cancer; PC, prostate cancer; BC, breast cancer; GC, gastric cancer; CML, chronic myelogenous leukemia; HCC, hepatocellular carcinoma.

micro-RNA	Cancer Type	Source	Cohort Size	Diagnostic	Prognosis	Reference
152 (−)	CRC	Tissue	28	+/−	−	[133]
152 (−)	BC invasive	Tissue	30	−	Poor	[134]
152 (−)	GC	Tissues	42	−	−	[137]
152 (+)	CML	Bone Marrow	40	-	-	[137]
152 (−)	HCC	Tissue	89	−	+/−	[138]
152 (−)	Stage I-IIIA NSCLC	Plasma	52	−	Reduced DFS	[142]
152 (−)	PC, lung, CRC, BC	Plasma	204	+	−	[145]
152 (−)	BC stage I-II	Plasma	106	+	−	[146]

**Table 7:** Elevated (+) miRNA-106b levels are shown, as are their target genes, their biological effects and whether these factors can sensitize cells to certain therapeutic agents. The cell types indicate the types of cells characterized. CD, Crohn’s Disease; CRC, colorectal cancer.

micro-RNA	Target	Positive Effect	Sensitizing Agent	Cell Type	Reference
106b (+)	RB	Reduced Cell Arrest	−	Laryngeal carcinoma HEP2G+T1U212	[147]
106b (+)	ATG16L1	Decreased Autophagy	−	CD	[148,149]
106b (+)	PTEN	Tumor Initiation Stemness	Radiation	CRC cell lines	[151]
106b (+)	p21 (indirectly)	Tumor Initiation Stemness	Radiation	CRC cell lines	[151]
106b (+)	DLC-1	EMT	−	CRC TissuesCRC cell lines	[152]
106b (+)	FAT4	Viability Angiogenesis Migration	−	CRC TissuesCRC cell lines	[153]

**Table 8:** Elevated (+) or reduced (−) miRNA-106b levels are shown, as are the cancer types, source of materials the miRNA was detected from and the patient cohort size. The negative or positive use of the technique in diagnostic or prognostic evaluation of patients are denoted by − or +, respectively. Exo, exosomal; RCCC, renal clear cell carcinoma; CC, colon cancer; CRC, colorectal cancer; OS, overall survival, DFS, disease-free survival; *, not statistically significant.

micro-RNA	Cancer Type	Source	Cohort Size	Diagnostic	Prognosis	Reference
106b (+) Exo	CRC	Serum	80	+	−	[152]
106b (−)	CC	Tissue	180	−	Long OS *	[156]
106b (+)	Metastatic CRC	Tissue	95	−	Short OS/DFS	[157]
106b (−)	RCCC	Tissue	27 studies	−	Poor	[158]
